# On the Treewidths of Graphs of Bounded Degree

**DOI:** 10.1371/journal.pone.0120880

**Published:** 2015-04-07

**Authors:** Yinglei Song, Menghong Yu

**Affiliations:** School of Electronics and Information Science Jiangsu University of Science and Technology Zhenjiang, Jiangsu 212003, China; Bangladesh University of Engineering and Technology, BANGLADESH

## Abstract

In this paper, we develop a new technique to study the treewidth of graphs with bounded degree. We show that the treewidth of a graph *G = (V, E)* with maximum vertex degree *d* is at most $\left(1-Ce^{-4.06\sqrt{d}})|V\right|$ for sufficiently large d, where *C* is a constant.

## Introduction

Tree decomposition is one of the most important concepts developed in graph theory in the past two decades. In a tree decomposition, graph vertices are grouped into vertex subsets, each of the vertex subsets is represented with a single node and all nodes are connected into a tree. Tree decomposition has provided original but profound insights into structural properties of graphs. For example, tree decomposition is the fundamental tool in the proof of the graph minor theorem [13, 14, 15, 16, 17, 18]. On the other hand, tree decomposition also has important applications in algorithm design and complexity research. A generic dynamic programming framework has been available for solving many NP-hard problems on graphs using tree decomposition [2]. Based on this framework, important algorithmic and complexity results have been found for some graph theoretic optimization problems [3, 9].

**Fig 1:**
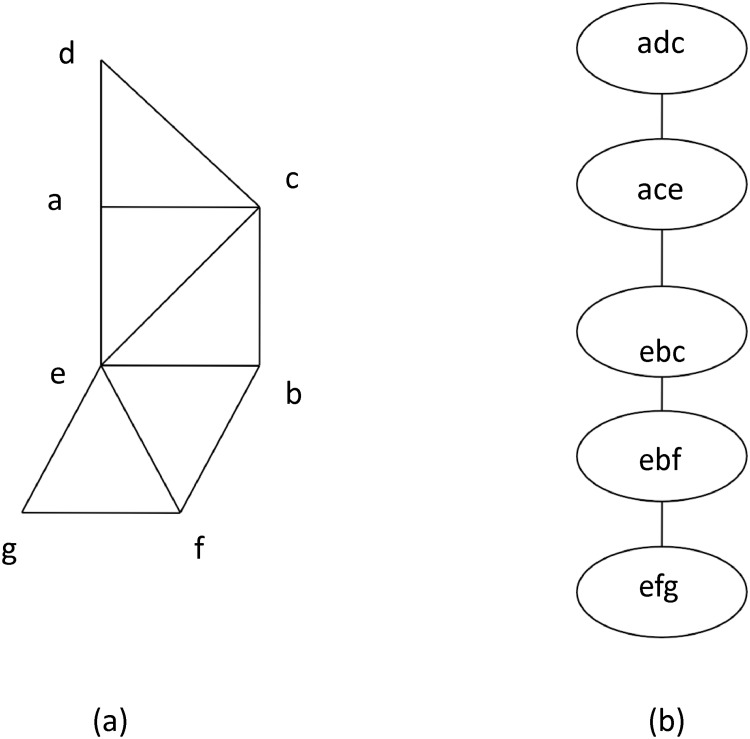
An example of graph tree decomposition. (a) a graph; (b) a tree decomposition of the graph in (a).

Fig. 1(b) shows a tree decomposition of the graph in Fig. 1(a). Tree decomposition provides a new topological view in the structure of a graph and has been shown to be related to many deep properties related to graph minors [14, 16]. These properties have played fundamental roles in the proof of the graph minor theorem. Moreover, tree decomposition also has important implications in algorithm design. For example, treewidth provides a structure parameter for a graph and many NP-hard graph optimization problems can be efficiently solved when the treewidth of the underlying graph is small [2]. More specifically, given a tree decomposition of a graph, we can easily identify subproblems for many NP-hard optimization problems and partial optimal solutions for these subproblems can be extended or combined with an exhaustive search performed only on vertices in a single tree node. As a result, given a tree decomposition, a dynamic programming approach can be employed to solve these optimization problems.

Treewidth is a structural parameter often used to evaluate a tree decomposition. The treewidth of a tree decomposition is determined by the maximum number of vertices contained in a single tree node and the treewidth of a graph is the minimum treewidth over all its tree decompositions. Based on the aforementioned dynamic programming framework, some NP-hard optimization problems including Maximum Independent Set and Minimum Dominating Set can be solved in time *O*(*f*(*t*)∣*V*∣), given a tree decomposition of treewidth *t* for the underlying graph *G* = (*V*, *E*) [2]. However, since *f*(*t*) is in general an exponential function of *t*, the treewidth of the available tree decomposition often determines the computational efficiency of this dynamic programming method.

A well known example is that, a maximum independent set in a graph *G* = (*V*, *E*) can be found in time *O*(2^*k*^∣*V*∣) based on a tree decomposition of treewidth *k* for *G* [2]. A more generic result states that, any graph optimization problem that can be formulated with Monadic Second Order (MSO) logic can be solved in polynomial time when a tree decomposition of bounded treewidth is available for the underlying graph [8]. Treewidth is often important for the computational efficiency of these algorithms in the sense that the most computationally intensive part in such an algorithm arises from an exponential function of the treewidth.

Computing the treewidth of a graph is an NP-hard problem [1]. Due to the difficulty of finding the treewidth of a graph, a few efficient algorithms and methods have been developed to estimate its upper bounds and lower bounds [4, 5, 6, 12]. For some graph families, an upper bound for treewidth can be estimated from other structural parameters with a simple formula. For example, the treewidth of a planar graph is bounded from above by 3*l* + 1 if the diameter of the graph is *l* [3, 9]. The treewidth of a graph with *m* edges has been shown to be less than *m*/5 [10]. In [7], an upper bound for treewidths are obtained for *k*-chordal graphs, where *k* is a constant. An improvement of this bound is recently provided in [11]. However, due to the difficulty of relating treewidth to other structural parameters, new tools are needed to obtain similar results for some graph families, such as graphs with bounded degree.

In this paper, we consider graphs of bounded degree and develop a nontrivial asymptotic upper bound for the treewidths of such graphs. Based on a new technique, **α* tree backbone*, we show that the treewidth of a graph with maximum vertex degree *d* is asymptotically bounded from above by $\left(1-Ce^{-4.06\sqrt{d}})|V\right|$ for large enough *d*, where *C* is a constant. The result shows that the technique of *α* tree backbone can be effectively used to provide new insights into the structural properties of graphs of bounded degree.

## Preliminaries

The graphs in this paper are undirected graphs without loops. For a given graph *G* = (*V*, *E*) and a vertex *v* ∈ *V*, *N*(*v*) is the set of vertices that are connected to *v* by an edge in *G* and ∣*N*(*v*)∣ is the *degree* of *v*. *N*[*v*] denotes {*v*} ∪ *N*(*v*). The *degree* of a graph is the maximum degree of all its vertices. To simplify the notation, we use *G* − *v* to represent the graph obtained by removing *v* and all the edges incident to *v* from *G*. For a vertex subset *U*, we use *G* − *U* to denote the graph obtained from *G* by removing all vertices in *U* and the edges incident to them from *G*. For a subset *U* ⊆ *V*, *N*(*U*) denotes the set of all vertices that are not in *u* but are joined by an edge to at least one vertex in *U*; *G*[*U*] is the subgraph induced by *U* in *G*. We use *d*(*G*) to denote the size of the minimum independent dominating set in graph *G*. A *path* in a graph is a sequence of vertices *v*_1_, *v*_2_, ⋯, *v*_*l*_ such that there is a graph edge between *v*_*i*_ and *v*_*i*+1_ (1 ≤ *i* < *l*). We use *P* = (*v*_1_, *v*_2_, ⋯, *v*_*l*_) to represent a path *P* of length *l*. A *cycle* of length *l* is a sequence of vertices *v*_1_, *v*_2_, ⋯, *v*_*l*_ such that there is a graph edge between *v*_*i*_ and *v*_(*i* + 1) *mod l*_.

**Definition 1** [13] Let *G* = (*V*, *E*) be a graph, where *V* is the set of vertices in *G*, *E* denotes the set of edges in *G*. The pair (*T*, *X*) is a *tree decomposition* of graph *G* if it satisfies the following conditions:

1. *T* = (*I*, *F*) defines a tree, the sets of vertices and edges in *T* are *I* and *F* respectively,
2. *X* = {*X*_*i*_∣*i* ∈ *I*, *X*_*i*_ ⊆ *V*}, and ∀*u* ∈ *V*, ∃*i* ∈ *I* such that *u* ∈ *X*_*i*_,
3. ∀(*u*, *v*) ∈ *E*, ∃*i* ∈ *I* such that *u* ∈ *X*_*i*_ and *v* ∈ *X*_*i*_,
4. ∀*i*, *j*, *k* ∈ *I*, if *k* is on the path that connects *i* and *j* in tree *T*, then *X*_*i*_ ∩ *X*_*j*_ ⊆ *X*_*k*_.

The *treewidth of the tree decomposition* (*T*, *X*) is defined as max_*i*∈*I*_ ∣*X*_*i*_∣ − 1. The *treewidth of the graph* *G* is the minimum treewidth over all possible tree decompositions of *G*.

**Definition 2** Let *G* = (*V*, *E*) be a graph, a vertex subset *F* ⊆ *V* is a *feedback vertex set* if graph *G* − *F* is acyclic. The *minimum feedback vertex set* in a *G* is the feedback vertex set with the minimum number of vertices.

## An Asymptotic Upper Bound for TreeWidth

**Lemma 1** If *F* is a feedback vertex set for graph *G* = (*V*, *E*), the treewidth of *G* is bounded by ∣*F*∣.

Proof. It is not difficult to see that since *G* − *F* is a tree, its treewidth is bounded by 1. Based on such a tree decomposition, we can simply include all vertices in *F* to every tree node in this tree decomposition and obtain a tree decomposition for *G*. The treewidth of this tree decomposition is bounded by ∣*F*∣.

Based on Lemma 3, an upper bound of the treewidth of a graph can be obtained from the size of a feedback vertex set in the graph. We focus on finding an upper bound for the minimum size of the feedback vertex set in a graph of degree bounded by *d*.

**Definition 3** For a graph *G* = (*V*, *E*) of degree *d*, an *α* *tree backbone* (0 < *α* < 1) of the graph is a subgraph *T* of *G* that satisfies the following:

1. *T* is a forest that contains a number of trees;
2. for each tree in *T* that contains more than 2 internal nodes, each internal node is of degree *d*;
3. for any vertex *v* of degree *d*, if *v* ∉ *T*, at least *αd* vertices in *N*(*v*) are nodes of the trees in *T*.

For a graph with maximum vertex degree *d*, an *α* tree backbone is a maximal forest such that each internal node in the forest is a vertex of degree *d* in *G* and has at least (1 − *α*)*d* child nodes in the tree. We prove in the following lemma that we are able to construct an *α* tree backbone in polynomial time.

**Lemma 2** For a graph *G* = (*V*, *E*) of degree *d*, given a positive number *α* < 1, there exists an algorithm that can find an *α* tree backbone *T* in polynomial time.

Proof. We show that *T* can be constructed by a greedy algorithm. Initially, we mark all vertices in *G* to be unselected. We arbitrarily choose an unselected vertex *v* of degree *d* and mark *v* and all vertices in *N*(*v*) as selected. We then proceed to vertices in *N*(*v*). For each vertex *u* ∈ *N*(*v*), all of the unselected neighbors of *u* are marked as selected if it is of degree *d* and the number of its unselected neighbors is *d* − 1. We recursively apply this procedure until we cannot mark more vertices as selected. We then include all selected vertices in *T*. For each remaining unselected vertex of degree *d*, we simply check the number of its neighbors that have been selected. If this number is smaller than *αd*, we include the vertex and its unselected neighbors in *T* and mark them as selected. It is not difficult to verify that this greedy algorithm can find an *α* tree backbone *T* in polynomial time.

For a given positive *α* < 1 and a graph *G* = (*V*, *E*) of degree at most *d*, based on an *α* tree backbone *T* of the graph, any vertex of degree *d* that is not in *T* has at least *αd* neighbors that are leaves of the trees in *T*. The following lemma considers only the vertices that are of degree *d* and not in *T*.

**Theorem 1** Given a graph *G* = (*V*, *E*) of maximum degree *d* and a positive number *α* < 1, if *T* is an *α* tree backbone in *G*, there exists a vertex subset *S* that satisfies the following:

1. A connected component in *G* − *S* is either a tree or a graph of degree bounded by *d* − 1;
2. $\left|S|\leq (\frac{2}{(1-\alpha )d}+\frac{2d-1+\sqrt{d}}{\alpha (\sqrt{d}+1)d})|V\right|$.

Proof. For a given *α* tree backbone *T* that contains *m* vertices, we consider the set of the internal nodes that are connected to a leaf in a tree in *T*. We denote such a set with *I*. We assume ∣*I*∣ = *s* and denote the nodes in *I* with *v*_1_, *v*_2_, ⋯, *v*_*s*_. In addition, we use *d*_1_, *d*_2_, ⋯, *d*_*s*_ to denote the degree of *v*_1_, *v*_2_, ⋯, *v*_*s*_ in *T*. It is not difficult to see that the following inequality holds.

$$\begin{array}{c} \sum_{i=1}^{s}d_{i}\leq 2\left(m-1\right)<2m \end{array}$$ (1)

The inequality holds since each edge is counted at most twice in the summation and there are at most *m* − 1 edges in *T*. On the other hand, since the degree of each node in *I* is at least (1 − *α*)*d*, we also have the following inequality.

$$\begin{array}{c} \sum_{i=1}^{s}d_{i}\geq \left(1-\alpha \right)ds \end{array}$$ (2)

Therefore, we can obtain

$$\begin{array}{c} s<\frac{2m}{(1-\alpha )d} \end{array}$$ (3)

We thus have $|I|<\frac{2m}{(1-\alpha )d}$. We now consider the vertices that are of degree *d* and not in *T*. We assume that such vertices form a vertex set *V*_1_ and their neighbors in *T* form vertex set *V*_2_. The bipartite subgraph formed between *V*_1_ and *V*_2_ is *H* = (*V*_1_ ∪ *V*_2_, *F*), such that for vertices *u* ∈ *V*_1_ and *v* ∈ *V*_2_, (*u*, *v*) ∈ *F* if and only if (*u*, *v*) ∈ *E*. The degree of vertices in *V*_1_ in *H* is at least *αd*. Note that we can assume the degree of each vertex in *V*_2_ in *H* is at most *d* − 1, since each such vertex is connected to at least one vertex in *T* by an edge and its degree is at most *d* in *G*.

We now show that *H* contains a dominating set *D* of size no larger than $\frac{2d-1+\sqrt{d}}{\alpha (\sqrt{d}+1)d}m$. To see this, we apply the following operations to vertices in *V*_2_. If *v* ∈ *V*_2_ and it’s degree in *H* is larger than a given positive number *c*, we simply include *v* in *D* and remove its neighbors in *V*_1_ from *H*. We repeat this until no remaining vertex in *V*_2_ have degree more than *c* in *H*, we then include all the remaining vertices in *V*_1_ in *D*.

It is not difficult to see that the number of vertices that are in *V*_2_ and included in *D* is at most ∣*V*_1_∣/(*c* + 1), for the remaining vertices in *V*_1_ since their degree is at least *αd* in graph *H*, we have *αd*∣*V*′∣ ≤ *c*∣*V*_2_∣, where *V*′ is the set of remaining vertices in *V*_1_. Combining the two we can obtain that the size of this dominating set is at most $\frac{\left|V_{1}\right|}{c+1}+\frac{c|V_{2}|}{\alpha d}$. On the other hand, since we have *αd*∣*V*_1_∣ ≤ (*d* − 1)∣*V*_2_∣, we get $\left|D|\leq \frac{d-1+c(c+1)}{(c+1)\alpha d}|V_{2}\right|$. Now if we let $c=\sqrt{d}$ and we obtain a dominating set with its size bounded by $\frac{2d-1+\sqrt{d}}{\alpha (\sqrt{d}+1)d}|V_{2}|$ from above.

We now consider the set *S* = *I* ∪ *D*, it is not difficult to see that after removing all vertices in *S*, each connected component in the resulting graph *G* − *S* is either a tree or a graph of degree bounded by *d* − 1. and we have $\left|S|\leq (\frac{2}{(1-\alpha )d}+\frac{\left(2d-1+\sqrt{d}\right)}{\alpha (\sqrt{d}+1)d})|V\right|$. The theorem has been proved.

Based on Theorem 1, we are able to compute an asymptotic upper bound for the size of the minimum feedback vertex set in a graph *G* with maximum vertex degree *d*, where *d* is an integer that is sufficiently large. Specifically, Theorem 1 states that we are able to remove a fraction of approximately $2/\sqrt{d}$ of the vertices from such a graph such that each connected component in the resulting graph is either a tree or a graph with its maximum degree bounded by *d* − 1. We can recursively apply this procedure to each connected component that is not a tree and we show that this procedure can lead to an upper bound estimate for the size of the minimum feedback vertex set.

**Lemma 3** Given a graph *G* = (*V*, *E*) of maximum degree *d*, there exists an independent vertex subset *S* ⊂ *V* such that ∣*S*∣ ≤ ∣*V*∣/2 and *G* − *S* is a graph of maximum degree *d* − 1.

Proof. To obtain such an *S*, we can arbitrarily choose a vertex *u* of degree *d* and include it in *S*. We then remove *u* and the edges incident to it from *G* and recursively apply the same procedure on the resulting graph until no such vertices can be found. *S* induces an independent set in *G* and we have ∣*E*∣ ≥ *d*∣*S*∣. On the other hand, ∣*E*∣ ≤ *d*∣*V*∣/2. This leads to ∣*S*∣ ≤ ∣*V*∣/2.

**Definition 4** Given a graph family *F* and an algorithm *A* that can compute a feedback vertex set in a graph in *F*, the *upper bound function* *u*(*F*, *A*) is defined as $max_{G\in F}\left\{\frac{B(G)}{\left|V\right|}\right\}$, where *G* = (*V*, *E*) is a graph in *F* and *B*(*G*) is the feedback vertex set computed by *A* in *G*.

We consider the family of graphs of maximum degree *d*. We use *L*(*d*) to denote the family. From Theorem 1 and Lemma 3, we are able to develop two algorithms that can compute a feedback vertex set for each graph in *L*(*d*). Indeed, after computing the set *S* as stated in Theorem 1 or Lemma 3 in a graph *G* = (*V*, *E*) ∈ *L*(*d*), each connected component in the resulting graph *G* − *S* is either a tree or a graph in *L*(*d* − 1). The algorithms terminate if *G* − *S* is empty or does not contain a connected component that is not a tree. Otherwise, each component that is not a tree in *G* − *S* is recursively processed. In the rest of the paper, we use *A*_1_ to denote the algorithm developed based on Theorem 1 and *A*_2_ for the algorithm developed based on Lemma 3.

**Theorem 2** For a given graph *G* = (*V*, *E*) of maximum degree *d*, there exists a positive constant *C* such that for a sufficiently large *d*, *G* contains a feed back vertex set with size at most $\left(1-Ce^{-4.06\sqrt{d}})|V\right|$. Such a feedback vertex set can be found in polynomial time.

Proof. From Theorem 1, we can let *α* = 0.99 and there exists an integer *D*_0_ such that when *d* > *D*_0_, we can find a vertex subset *S* ⊆ *V* that satisfies the property that each connected component of *G* − *S* is either a tree or a graph with maximum degree of *d* − 1 and $|S|\leq 2.021|V|/\sqrt{d}$. An additional requirement for selecting *D*_0_ is specified later in the proof.

Based on *D*_0_, the following algorithm *A*_3_ can compute a feedback vertex set in a graph *G* = (*V*, *E*).

1. For each connected component *M* that is not a tree in *G* and does not contain a vertex of degree larger than *D*_0_, apply *A*_2_ to *M* to get a feedback vertex set *F* in *M* and include all vertices in *F* into the feedback vertex set;
2. the algorithm returns if *G* does not contain a connected component that is not a tree and contains at least one vertex of degree larger than *D*_0_. Otherwise it continues to step 3;
3. for each connected component *E* that is not a tree in *G* and contains at least one vertex of degree larger than *D*_0_, find a subset *S* in *E* as described in the proof of Theorem 1, include all vertices in *S* into the feedback vertex set and remove *S* from *E*;
4. set the resulting graph to be *G*′ and recursively apply the algorithm to *G*′;

From Definition 4, a graph *G* = (*V*, *E*) with maximum degree *d* has a feedback vertex set of size at most *u*(*L*(*d*), *A*_3_)∣*V*∣. From the above description of *A*_3_, we obtain *u*(*L*(*D*_0_), *A*_3_) = *u*(*L*(*D*_0_), *A*_1_). *A*_3_ thus returns a feedback vertex set of size at most *u*(*L*(*D*_0_), *A*_1_)∣*V*∣ in a graph *G* = (*V*, *E*) with maximum degree *D*_0_. From Lemma 3, we immediately obtain that *u*(*L*(*d*), *A*_1_) must satisfy *u*(*L*(*d*), *A*_1_) ≤ (*u*(*L*(*d* − 1), *A*_1_) + 1)/2. If we consider the case where *d* = 2, we find that $u(L(2),A_{1})\leq \frac{1}{2}$. For a finite integer *D*_0_, it is obvious that *u*(*L*(*D*_0_), *A*_1_) is a positive constant strictly less than 1. Since *u*(*L*(*D*_0_), *A*_3_) = *u*(*L*(*D*_0_), *A*_1_), *u*(*L*(*D*_0_), *A*_3_) is also a positive constant strictly less than 1.

We assume *A*_3_ returns a feedback vertex set of size *U*(*L*(*d*), *A*_3_)∣*V*_1_∣ in graph *G*_1_ = (*V*_1_, *E*_1_) ∈ *L*(*d*). From Theorem 1, it is clear that when *d* > *D*_0_, we can find a vertex subset *S*_1_ ⊆ *V*_1_ such that each connected component of *G*_1_ − *S*_1_ is either a tree or a graph with maximum degree of *d* − 1 and $|S_{1}|\leq 2.021|V_{1}|/\sqrt{d}$. We use *F*(*G*_1_ − *S*_1_) to denote the feedback vertex set obtained by *A*_3_ on *G*_1_ − *S*_1_. Since each connected component of *G*_1_ − *S*_1_ is either a tree or a graph in *L*(*d* − 1), it is not difficult to see that the following inequality holds

$$\begin{array}{c} |F\left(G_{1}-S_{1}\right)|\leq u\left(L\left(d-1\right),A_{3}\right)\left(\right|V_{1}\left|-\right|S_{1}\left|\right) \end{array}$$ (4)

On the other hand, since *A*_3_ returns *S*_1_ ∪ *F*(*G*_1_ − *S*_1_) as a feedback vertex set in *G*_1_, we have

$$\begin{array}{ccc} u\left(L\left(d\right),A_{3}\right)\left|V_{1}\right| & = & |F\left(G_{1}-S_{1}\right)\left|+\right|S_{1}| \end{array}$$ (5)

$$\begin{array}{ccc} & \leq & u\left(L\left(d-1\right),A_{3}\right)\left(\right|V_{1}\left|-\right|S_{1}\left|)+\right|S_{1}| \end{array}$$ (6)

$$\begin{array}{ccc} & = & \left(1-u\left(L\left(d-1\right),A_{3}\right)\right)|S_{1}|+u\left(L\left(d-1\right),A_{3}\right)\left|V_{1}\right| \end{array}$$ (7)

Since *u*(*L*(*d* − 1), *A*_3_) ≤ 1 and $|S_{1}|\leq 2.021|V_{1}|/\sqrt{d}$, we can immediately obtain the following recursion relation for *u*(*L*(*d*), *A*_3_) when *d* > *D*_0_.

$$\begin{array}{c} u\left(L\left(d\right),A_{3}\right)\leq \frac{2.021}{\sqrt{d}}\left(1-u\left(L\left(d-1\right),A_{3}\right)\right)+u\left(L\left(d-1\right),A_{3}\right) \end{array}$$ (8)

Since the following relation holds for a sufficiently small positive number *x*

$$\begin{array}{c} 1-x\geq e^{-1.004x} \end{array}$$ (9)

it is clear that we can select sufficiently large *D*_0_ such that when *d* > *D*_0_, we have

$$\begin{array}{c} 1-\frac{2.021}{\sqrt{d+1}}\geq e^{-\frac{2.03}{\sqrt{d+1}}} \end{array}$$ (10)

On the other hand, we have

$$\begin{array}{ccc} \frac{2.03}{\sqrt{d+1}} & < & \frac{4.06}{\sqrt{d+1}+\sqrt{d}} \end{array}$$ (11)

$$\begin{array}{ccc} & = & 4.06(\sqrt{d+1}-\sqrt{d}) \end{array}$$ (12)

We can thus obtain

$$\begin{array}{c} 1-\frac{2.021}{\sqrt{d+1}}\geq e^{-4.06(\sqrt{d+1}-\sqrt{d})} \end{array}$$ (13)

We can choose an appropriate constant *C* that satisfies $u(L(D_{0}),A_{3})\leq 1-Ce^{-4.06\sqrt{D_{0}}}$. We next show that $u(L(d),A_{3})\leq (1-Ce^{-4.06\sqrt{d}})$ when *d* ≥ *D*_0_. Based on the principle of induction, when *d* = *D*_0_ the relation holds due to the selection of constant *C*. Now, we assume that the relation holds when *d* = *l*, based on the recursion relation, we have

$$\begin{array}{ccc} u(L\left(l+1\right),A_{3}) & \leq & \left(1-u\left(L\left(l\right),A_{3}\right)\right)\left(1-e^{-4.06(\sqrt{l+1}-\sqrt{l})}\right)+u\left(L\left(l\right),A_{3}\right) \end{array}$$ (14)

$$\begin{array}{ccc} & \leq & 1-e^{-4.06(\sqrt{l+1}-\sqrt{l})}\left(1-u\left(L\left(l\right),A_{3}\right)\right) \end{array}$$ (15)

$$\begin{array}{ccc} & \leq & 1-Ce^{-4.06\sqrt{l+1}} \end{array}$$ (16)

Based on the principle of induction, we know *u*(*L*(*d*), *A*_3_) is at most $1-Ce^{-4.06\sqrt{d}}$ when *d* ≥ *D*_0_. In addition, from Lemma 2, *A*_3_ needs polynomial time to find such a feedback vertex set.

Here, we need to point out that, based on Lemma 3, we can also construct a recursive relation for *A*_2_. However, this recursion can only lead to an upper bound estimation of (1 − 1/2^*O*(*d*)^)∣*V*∣. This trivial asymptotic upper bound now has been significantly improved using the technique of *α* tree backbone. Based on the relation between the treewidth and the size of a minimum feedback vertex set, we also obtain an asymptotic upper bound for the treewidth of a graph with bounded maximum vertex degree.

**Corollary 1** For a given graph *G* = (*V*, *E*) with maximum vertex degree *d*, there exists a positive constant *C* such that for a large enough *d*, the treewidth of the graph is bounded from above by $\left(1-Ce^{-4.06\sqrt{d}})|V\right|$.

Theorem 2 also provides an asymptotic upper bound for the treewidths of sparse graphs. Specifically, for a graph *G* = (*V*, *E*), where ∣*E*∣ ≤ Δ∣*V*∣ and Δ is a positive constant independent of *G*, we have the following theorem.

**Theorem 3** Given a graph *G* = (*V*, *E*), where ∣*E*∣ ≤ Δ∣*V*∣ and Δ is a positive constant independent of *G*, there exists a positive constant *C*′ such that for a large enough Δ, the treewidth of *G* is bounded from above by $\left(1-C^{\prime }e^{-8.12\sqrt{\Delta }})|V\right|$.

Proof. First, since ∣*E*∣ ≤ Δ∣*V*∣, the number of vertices with degree larger than 4Δ is at most ∣*V*∣/2. We assume the number of such vertices is *β*∣*V*∣ and we have 0 ≤ *β* ≤ 1/2. Now, we remove all these vertices and edges incident to them from *G* and the resulting graph *G*′ is a graph with maximum vertex degree 4Δ. Apply Theorem 2 to graph *G*′, for large enough Δ, we can obtain a tree decomposition *T*′ for *G*′ with its treewidth at most $\left(1-Ce^{-8.12\sqrt{\Delta }})|V^{\prime }\right|$, where *C* is a positive constant. We can thus obtain a tree decomposition *T* for *G* by including the removed vertices in each tree node of *T*′. The tree width of *T* is at most $\beta |V|+(1-Ce^{-8.12\sqrt{\Delta }})(1-\beta )|V|$. Since *β* ≤ 1/2, the treewidth of *T* is bounded from above by $\left(1-\frac{C}{2}e^{-8.12\sqrt{\Delta }})|V\right|$.

## Conclusions

In this paper, we provided an original perspective on the structure of a graph with bounded degree. We develop a new technique, *α* tree backbone, to analyze the treewidth for graphs of bounded degree. Our analysis leads to a nontrivial asymptotic upper bound for the treewidth of such graphs.

We have seen in the proof of Theorem 1 that the size of the dominating set *D* in the bipartite graph *H* is crucial for our analysis and improvements made on its size can lead to further improved bounds for treewidth. In particular, we conjecture that the treewidth of a graph *G* = (*V*, *E*) with maximum vertex degree *d* is in fact asymptotically bounded by $\left(1-\frac{C}{d^{c}})|V\right|$, where *C* and *c* are constants. However, the technique based on *α* tree backbone may not be sufficient to obtain such an upper bound. Our future work may focus on providing a proof or counterexample for this conjecture.
